# Water Level Reconstruction and Prediction Based on Space-Borne Sensors: A Case Study in the Mekong and Yangtze River Basins

**DOI:** 10.3390/s18093076

**Published:** 2018-09-13

**Authors:** Qing He, Hok Sum Fok, Qiang Chen, Kwok Pan Chun

**Affiliations:** 1 School of Geodesy and Geomatics, Wuhan University, Wuhan 430079, China; heqing@whu.edu.cn; 2 Key Laboratory of Geospace Environment and Geodesy, Ministry of Education, Wuhan University, Wuhan 430079, China; 3 Geophysics Laboratory, Faculty of Science, Technology and Communication, University of Luxembourg, 2, Avenue de l’Université, L-4365 Esch-sur-Alzette, Luxembourg; 4 Department of Geography, Hong Kong Baptist University, Hong Kong, China; kpchun@hkbu.edu.hk

**Keywords:** water level, TRMM, GRACE Drought Severity Index (DSI), TRMM-based Standardized Precipitation Index (SPI), Mekong River Basin, Yangtze River Basin

## Abstract

Water level (WL) measurements denote surface conditions that are useful for monitoring hydrological extremes, such as droughts and floods, which both affect agricultural productivity and regional development. Due to spatially sparse in situ hydrological stations, remote sensing measurements that capture localized instantaneous responses have recently been demonstrated to be a viable alternative to WL monitoring. Despite a relatively good correlation with WL, a traditional passive remote sensing derived WL is reconstructed from nearby remotely sensed surface conditions that do not consider the remotely sensed hydrological variables of a whole river basin. This method’s accuracy is also limited. Therefore, a method based on basin-averaged, remotely sensed precipitation from the Tropical Rainfall Measuring Mission (TRMM) and gravimetrically derived terrestrial water storage (TWS) from the Gravity Recovery and Climate Experiment (GRACE) is proposed for WL reconstruction in the Yangtze and Mekong River basins in this study. This study examines the WL reconstruction performance from these two remotely sensed hydrological variables and their corresponding drought indices (i.e., TRMM Standardized Precipitation Index (TRMM-SPI) and GRACE Drought Severity Index (GRACE-DSI)) on a monthly temporal scale. A weighting procedure is also developed to explore a further potential improvement in the WL reconstruction. We found that the reconstructed WL derived from the hydrological variables compares well to the observed WL. The derived drought indices perform even better than those of their corresponding hydrological variables. The indices’ performance rate is owed to their ability to bypass the influence of El Niño Southern Oscillation (ENSO) events in a standardized form and their basin-wide integrated information. In general, all performance indicators (i.e., the Pearson Correlation Coefficient (PCC), Root-mean-squares error (RMSE), and Nash–Sutcliffe model efficiency coefficient (NSE)) reveal that the remotely sensed hydrological variables (and their corresponding drought indices) are better alternatives compared with traditional remote sensing indices (e.g., Normalized Difference Vegetation Index (NDVI)), despite different geographical regions. In addition, almost all results are substantially improved by the weighted averaging procedure. The most accurate WL reconstruction is derived from a weighted TRMM-SPI for the Mekong (and Yangtze River basins) and displays a PCC of 0.98 (and 0.95), a RMSE of 0.19 m (and 0.85 m), and a NSE of 0.95 (and 0.89); by comparison, the remote sensing variables showed less accurate results (PCC of 0.88 (and 0.82), RMSE of 0.41 m (and 1.48 m), and NSE of 0.78 (and 0.67)) for its inferred WL. Additionally, regardless of weighting, GRACE-DSI displays a comparable performance. An external assessment also shows similar results. This finding indicates that the combined usage of remotely sensed hydrological variables in a standardized form and the weighted averaging procedure could lead to an improvement in WL reconstructions for river basins affected by ENSO events and hydrological extremes.

## 1. Introduction

Measuring the spatio-temporal water level (WL) in rivers, lakes, wetlands and reservoirs is of the utmost importance for increasing water usage efficiency, monitoring floods or droughts, and, hence, reducing agricultural and economic losses within a catchment [1,2]. In addition, other important hydrological variables, such as discharge, can also be obtained using a WL based on a stage-discharge rating curve (e.g., [3,4,5]). Therefore, a continuous WL time series is essential for enriching hydrological data and monitoring hydrological extremes within a river basin [6].

Traditionally, the WL has been measured by in situ hydrological stations, which are distributed unevenly and sparsely around the world and are dependent on the economy, politics, and geography of each nation [7]. Consequently, an alternative yielding continuous direct or indirect WL measurements must be sought. Satellite remote sensing has been demonstrated as a promising alternative for globally continuous monitoring of WL data, both directly and indirectly [8]. While altimetry satellites can directly measure the WL in large rivers (e.g., [9,10,11,12]), inland radar altimetry footprint signals are inevitably contaminated by lands around rivers that reduce the quality of altimetry measurements [13]. Traditional indirect WL monitoring is generally based on an empirical rating curve between the WL and an inundated area using high-resolution satellite images (e.g., [14,15]). A linear relationship between the WL and the surface area changes of a surface reservoir has also been applied (e.g., [16]). However, the relationship varies across different segments of the river, and it also changes over time in the same place due to natural factors, such as erosion and sedimentation [17,18]. The instability of the relationship thus reduces the accuracy of the WL reconstruction. Other localized remote sensing measurements, such as the Normalized Difference Vegetation Index (NDVI) [19] and Land Surface Temperature (LST) [20], have also been utilized during the past several decades. However, these instantaneous responses only represent the localized phenomena within a hydrological cycle, which contains combined signals with several sources of uncertainty [21]. For instance, Omute et al. [22] found that the NDVI is more sensitive to drought than to the WL, although it can capture the WL one-month in advance.

To achieve higher accuracy, remotely sensed data representing causal phenomena of hydrological cycle components (i.e., precipitation, evaporation or evapotranspiration, and terrestrial water storage (TWS)) in a river system can be adopted for the WL reconstruction. These data can potentially be better predictors of WL variations. For instance, the Tropical Rainfall Measuring Mission (TRMM) satellite has been demonstrated to be a reliable resource for monthly precipitation data in recent decades (e.g., [23,24,25]), but most of studies that have used it are limited to drought monitoring (e.g., [26,27]) and TRMM products evaluation (e.g., [28,29,30]). Gravity Recovery and Climate Experiment (GRACE) satellite data has been widely used to obtain TWS variations (e.g., [31,32]) and their successful assimilation into hydrological models (e.g., [33,34]) for various river basins. Therefore, remotely sensed precipitation and TWS data can be excellent choices for the WL reconstructions.

In fact, different empirical relationships between WL and precipitation based on ground observations and remote sensing data have been established in recent years [35,36,37]. For instance, significant correlations between the WL and the precipitation were found no matter in the upper, middle and lower Chalk of southern England [35]. Jiang et al. [36] found that a higher summer precipitation increased the summer runoff rate in the lower Yangtze River Basin between 1961 and 2000. Awange et al. [37] found that 80% of the WL falling in Lake Victoria was directly related to precipitation levels.

Several researchers qualitatively inferred that WL variations were linked to the TWS (e.g., [38]), but few studies quantitatively yielded an empirical or analytical formula for such a relationship, let alone a prediction. Frappart et al. [39] measured the water storage fluctuations from an altimetric-derived WL combined with Synthetic Aperture Radar (SAR) images in the Negro River Basin. Zhang et al. [40] established a rating curve to monitor the seasonal and annual water storage fluctuations of Dongting Lake by correlating the altimetric-derived WL with the observed water storage. In summary, the relationships between the WL and both precipitation and water storage have been scientifically investigated, but a practical usage of the abovementioned relationships is rarely explored, let alone the influence of climatic events, such as ENSO and monsoons, on WL reconstructions and predictions.

Drought indices, such as the Standardized Precipitation Index (SPI), are derived through the standardization process to depict the drought conditions [41] whether using remotely sensed or ground-based observed precipitation. Similarly, the GRACE Drought Severity Index (GRACE-DSI), newly developed by Zhao et al. [42], can also capture the spatio-temporal evolution of drought events. However, their usages for WL reconstructions and estimations have not been explored to date. Therefore, a standardized procedure focusing on reducing the influences of abnormal anomalies (e.g., climatic events) was employed to convert the TRMM precipitation and GRACE TWS into their corresponding drought indices (i.e., TRMM-SPI and GRACE-DSI)) followed by estimating the WL in the Mekong and Yangtze River basins.

This study aims to reconstruct the WL based on precipitation, TWS, and their related drought indices (i.e., TRMM-SPI and GRACE-DSI) for the Mekong and Yangtze River basins. Both hydrological regimes are mainly influenced by monsoons and ENSO events, making them suitable geographic regions for our study. In addition, a weighting factor is introduced to further improve the performance of WL reconstruction. Finally, three kinds of performance metrics, the Pearson Correlation Coefficient (PCC), Root-mean-squares error (RMSE), and Nash–Sutcliffe model efficiency coefficient (NSE) are used to evaluate all the WL estimations. The remotely sensed NDVI and LST serve as vehicles for a comparative analysis with the proposed WL reconstruction method, which is based on remote-sensed hydrological variables and their corresponding drought indices.

## 2. Geographical Setting of the Mekong River Basin (MRB) and the Yangtze River Basin (YRB)

Both the Mekong and Yangtze rivers originate from the three-rivers region (TRR) headwater source located at the southeastern Qinghai-Tibet Plateau. Both rivers exhibit apparent differences in terms of their geographical locations, as well as those of their monsoonal climate and its impact on the WL and the discharge volume. Their abovementioned characteristics make them the two ideal regions for our study.

**Figure 1:**
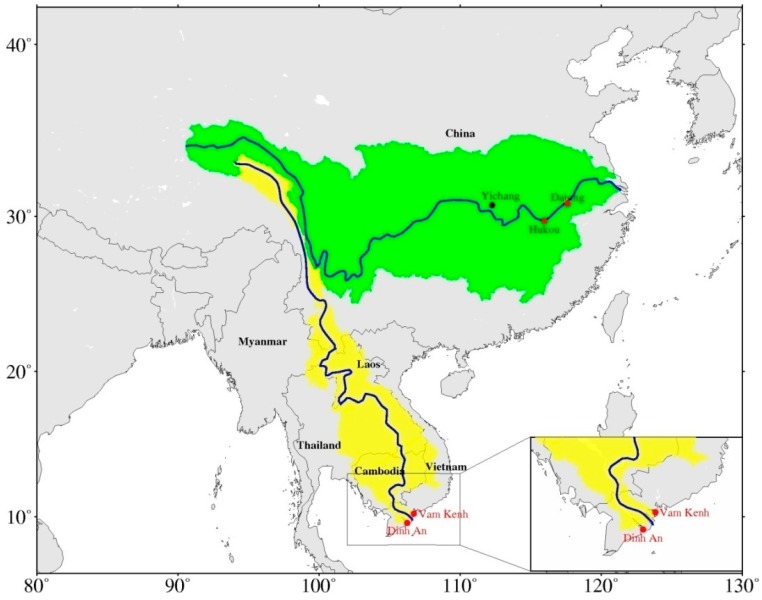
Map of the Yangtze River Basin (green region) and the Mekong River basin (yellow region) with selected stations in red dots located near the river estuary in this study.

The Mekong River, having a total length of 4909 km, is the 12th-longest river in the world [43]. This river’s extensive basin covers six southeast Asian countries with an area of 795,000 km^2^ (Figure 1). From the headwater source, the water flows from subtropical (i.e., TRR) to tropical regions (i.e., South China Sea (SCS)) through China, Burma, the Lao People’s Democratic Republic (Lao PDR), Thailand, Cambodia and Vietnam [44].

The Mekong River Basin (MRB) shows a typical monsoon climate, especially in the lower MRB located in the intersection zones of three types of monsoons (i.e., Indian Summer Monsoon (ISM), Western North Pacific Summer Monsoon (WNPSM), and East Asian Summer Monsoon (EASM)) [45], which are defined as the Asian-Pacific monsoon system by Wang and Lin [46]. Given the influence of monsoons, the middle and lower MRB have distinct rainy and dry seasons. The southwest monsoon from the ocean initiates the rainy season in May, reaches its peak in September and ends in October each year [47]. During that time, it contributes to 85% of the basin’s annual rainfall [47]. Less precipitation is observed during the dry seasons that occur from November to March of the following year [48]. The runoff recharge of the Mekong River in dry seasons is mainly driven by the water melting from the TRR.

The Yangtze River, the longest river in China and the third-longest river in the world, originates from the Tibetan Plateau and flows approximately 6300 km eastwards into the East China Sea [49,50] (Figure 1). The whole Yangtze River Basin (YRB), with an area of around 1,800,000 km^2^ [51], can be divided into three parts: The upper reach (between the headwater and Yichang), the middle reach (between Yichang and Hukou), and the lower reach (below the Hukou) [52,53]. The YRB is in the mid-latitudinal region of approximately 32°N, where the climate is subtropical and temperate [54].

The Asian-Pacific monsoon system also maintains a substantial influence over the entire YRB [55]. The ISM and the EASM in the Asian-Pacific system affect the precipitation in the upper and middle-lower YRB, respectively [50,56]. Thus, the mean annual precipitation varies from 270–500 mm in the upper YRB to 1600–1900 mm in the middle and lower YRB [57]. Under the monsoonal climate, the rainy season ranges from April to September, generating tremendous precipitation in the YRB, especially during mid-June to July (i.e., the plum rain season) [54].

In summary, the two basins are under the control of the Asian-Pacific monsoon system. The intensities of ISM, WNPSM and EASM are demonstrably affected by El Niño–Southern Oscillation (ENSO) events [58]. For example, the ISM, WNPSM and EASM are weakened during El Niño events (the warm phase of ENSO), leading to less precipitation in some regions [59,60]. Therefore, ENSO events have an important influence on the hydrological conditions in both the MRB and YRB.

## 3. Data Description

### 3.1. Ground-Based Observation Data

**Figure 2:**
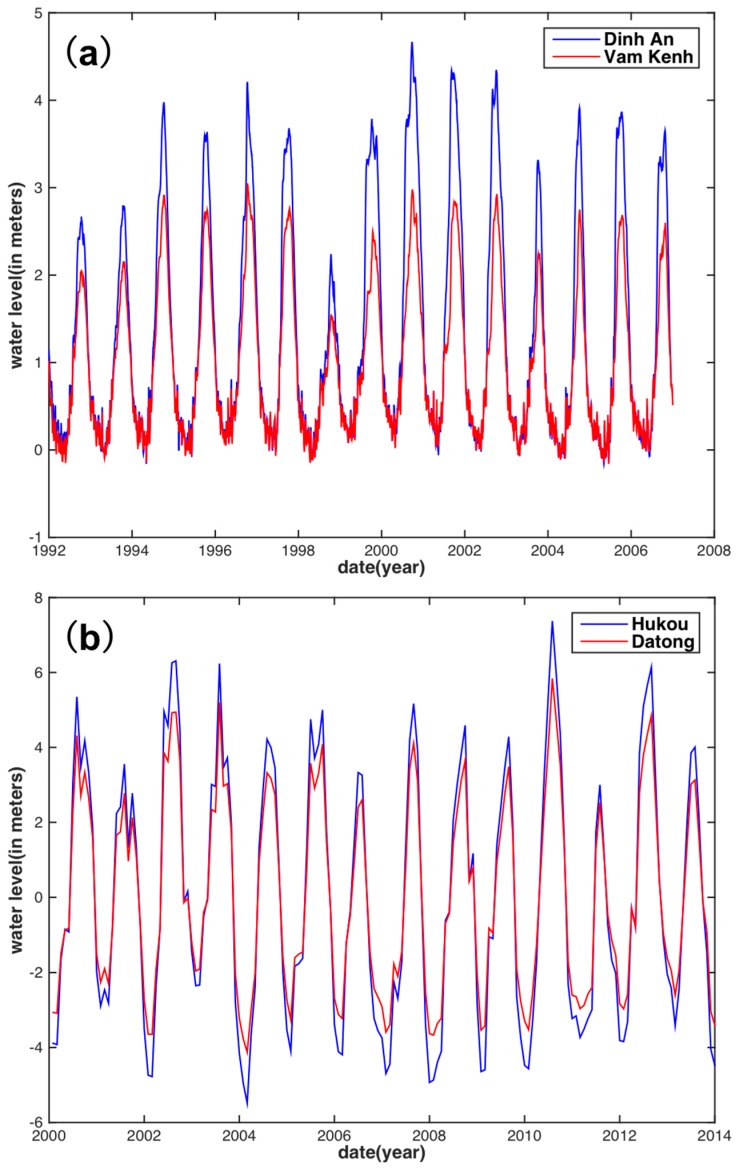
(**a**) Time series of water level at Dinh An (blue) station and Vam Kenh (red) station in the Mekong River Basin (MRB); (**b**) Time series of water level in Hukou (blue) station and Datong (red) station in the Yangtze River Basin (YRB).

Since basin-averaged TRMM precipitation and TWS were applied to reconstruct the whole basin WL, an in situ WL time series of the Vam Kenh and Dinh An stations located near the estuary mouth of the MRB were selected to reconstruct and validate the WL, respectively, in this study (Figure 1). These time series, with data spanning from 1 January 1992 to 31 December 2006, are available on request at the website of Mekong River Commission (http://www.mrcmekong.org). Figure 2a shows that the WL time series in both stations display a strong seasonality and are similar to each other despite differences in amplitudes. The annual maximum WL usually occurs in September resulting from the southwest monsoon, whereas the minimum WL happens in April in accordance with the general description in Section 2.

Since no station exists in the estuary mouth of the YRB because of oceanic tidal effects, the closest (Datong station) and next-closest stations (Hukou station) to the estuary mouth were chosen to reconstruct and validate the reconstructed WL, respectively. The WL time series of the two stations, with a time span between January 2000 and December 2013, have been obtained from the Changjiang Water Resources Commission, Ministry of Water Resources (http://www.cjh.com.cn). Figure 2b shows that the WL variations in the Datong and Hukou stations share the same pattern. The highest and lowest WLs usually occur in July and February, respectively, which are different from that of the MRB.

### 3.2. Spaceborne Data and Their Corresponding Derived Drought Indices

#### 3.2.1. TRMM Precipitation and Its Corresponding Drought Index (TRMM-SPI)

The launch of the Tropical Rainfall Measuring Mission (TRMM) satellite enabled us to monitor the spatio-temporal precipitation variations in the tropical and the subtropical regions [61]. Monthly 3B43 V7 precipitation products, with a spatial resolution of 0.25° and a coverage of latitude 50° N to 50° S validated by the TRMM Multi-satellite Precipitation Analysis (TMPA) [62], were used to either directly correlate with the observed WL or to derive the SPI followed by correlating it with the observed WL in this study. These data can be obtained from the NASA’s Goddard Earth Sciences and Data and Information Service Center (GES DISC) (https://disc.gsfc.nasa.gov/TRMM).

The SPI, developed by McKee et al. [41], was used to quantify the precipitation deficit on multiple time scales. It indicates the departure degree of the accumulative precipitation of a certain period related to a selected time scale with respect to normal conditions [63]. Compared to other widely used drought indices (e.g., PDSI) that consider various localized hydrological and meteorological inputs, SPI can be simply calculated by precipitation only, making it an effective index for monitoring the drought conditions in regions where climatic and hydrological information is scarce [64].

According to the initial definition of SPI by McKee et al. [41], it can be regarded as the standardized form of precipitation.

(1) $$\;SPI_{i,j}=\frac{P_{i,j}-\langle P_{j}\rangle }{\sigma_{j}}\;$$

where $P_{i,j}$, $\langle P_{j}\rangle$, and $\sigma_{j}$ are the precipitation in year i and month j, the mean precipitation in month j, and the standard deviation of precipitation for month j, respectively. The standardized form, displayed in Equation (1), should have a normal distribution. The log-normal and in particular the gamma distributions are the two commonly employed distributions in this case [63].

Lloyd-Hughes and Saunders [64] introduced a detailed calculation of SPI based on the log-normal and the gamma distributions. Taking a logarithm of precipitation for Equation (1), the SPI based on the log-normal distribution becomes,

(2) $$\;SPI_{i,j}=\frac{\ln(P_{i,j})-\langle \ln\left(P_{j}\right)\rangle }{\hat{\sigma_{j}}}\;$$

where the $\hat{\sigma_{j}}$ represents the sampled standard deviation of log-transformed precipitation.

Alternatively, the SPI based on gamma distribution can be obtained through the standardization of cumulative probability distribution. The cumulative probability distribution H(P) is derived as

(3)  H(P)=q+(1−q)G(P) 

where q is the probability of zero precipitation. The gamma probability density function (G(P)) is calculated as

(4) $$\;G\left(P\right)=\frac{1}{\beta^{\alpha }\tau \left(\alpha \right)}\overset{P}{\underset{0}{\int }}P^{\alpha -1}e^{-P/\beta }dP\;\;\;for\;P>0\;$$

where α>0 is a shape parameter, β>0 is a scale parameter, and P represents the precipitation accumulated in the selected time scale. τ(α) is the gamma function.

Multiple time-scaled SPI is a useful drought index that quantifies different types of drought events. For instance, the SPI on scales of 2–6 months can best capture the river discharge; the ground WL can be illustrated well by a 5–24 months time-scaled SPI; and the information of agricultural drought can be best described by the SPI with time scales of 2–3 months [65,66]. Owing to its applicability, the SPI based on gamma distribution was adopted and calculated using the TRMM monthly precipitation data product. Note that TRMM data time spans between 2000 and 2013 were used, while a 30-year continuous precipitation time series is desirable for a long-period signal.

#### 3.2.2. GRACE Terrestrial Water Storage (TWS) and Its Corresponding Drought Index (GRACE-DSI)

The launch of the Gravity Recovery and Climate Experiment (GRACE) enabled us to compute a global monthly Equivalent Water Height (EWH) (i.e., TWS) with a spatial resolution of 3° from the time-variable gravity observations (e.g., [67,68]). The degree-60 GRACE Level-2 Release 05 (RL05) monthly gravity data products, in the form of spherical harmonic coefficients (SHC), allow us to compute the EWH at a regular grid. These data can be obtained from GeoForschungsZentrum (GFZ) (ftp://rz-vm152.gfz-potsdam.de/grace/). The choice of GFZ L2 products is made because the calibrated uncertainties of the spherical harmonics coefficients (SHCs) are available [69]. The time span of the SHCs covered January 2003 to December 2013, with missing SHCs for January 2004, January 2011, June 2011, May 2012, October 2012. According to the result from Swenson et al. [70], the degree-one coefficients were added to account for the geocenter motion, whereas the C_20_ term was replaced in the GRACE data by the Satellite Laser Ranging (SLR) results [71] before deriving EWH. To lower uncertainties of EWH at a higher degree resulting from spatially correlated errors [72], a destriping process [73] and Gaussian filtering with a radius of 350 km were applied [74]. After these pre-processing procedures, monthly EWHs were used to either directly correlate with the ground-based observed WL or to derive the corresponding drought index (called GRACE Drought Severity Index (GRACE-DSI)) followed by correlating with the observed WL in this study.

GRACE-DSI is a newly proposed standardized drought severity index based on the GRACE-derived TWS, which can be classified into an 11-level of drought magnitude [42]. It exhibits favorable agreement with the PDSI, U.S. Drought Monitor (USDM) and NDVI. GRACE-DSI should capture deeper water storage changes when compared to the aforementioned traditional drought indices. To resist abnormal TWS anomalies that creates biases, this study uses the median of monthly TWS anomalies, in contrast to that of the mean of TWS anomalies proposed by Zhao et al. [42]. In addition, the median was used due to its robustness for extreme values while computing the monthly normal value [16]. Therefore, the GRACE-DSI is modified as

(5) $$\;I_{i,j}^{G}=\frac{S_{i,j}-med\left(S_{j}\right)}{s_{j}}\;$$

where $I_{i,j}^{G}$. and $S_{i,j}$ represents the GRACE-DSI and TWS in year i and month j, respectively. The $med\left(S_{j}\right)$ and $s_{j}$ components are the median and the sampled standard deviation of TWS for month j, separately.

## 4. Methodology and Assessment Scheme

### 4.1. Methodology

Because the observed WL near the estuary in this study represents the total input from the accumulated precipitation (TWS) of the whole river basin, the time series for the whole MRB and YRB are averaged or weight-averaged. This process is then followed by a linear regression analysis for establishing a correlative relationship between the observed WL and the two hydrological variables (i.e., precipitation and GRACE TWS) as well as their corresponding derived indices (i.e., TRMM-SPI and GRACE-DSI). The overlapping period for the two hydrological variables and their corresponding derived indices was from January 2000 to December 2006 (precipitation fitting with the WL in MRB), from January 2000 to December 2013 (precipitation fitting with the WL in YRB), from January 2003 to December 2006 (TWS fitting with the WL in MRB), and from January 2000 to December 2013 (TWS fitting with the WL in YRB), respectively.

These indices are normally obtained through standardization. Therefore, the observed WL of two basins was standardized to be consistent with the standardized forms of precipitation and TWS (i.e., TRMM-SPI and GRACE-DSI) before establishing their correlative relationship. The standardized procedure can be achieved by subtracting the monthly averaged WL from the median values of the corresponding month (i.e., the WL anomaly) for the entire overlapping time span divided by the standard deviation of the corresponding month, which can be formulated as

(6) $$\;h_{i,j}^{s}=\frac{h_{i,j}-med\left(h_{j}\right)}{s_{j}}\;$$

where $h_{i,j}^{s}$ and $h_{i,j}$ represents the standardized water level (SWL) and observed WL in year i and month j, respectively. The $med\left(h_{j}\right)$ and $s_{j}$ components are the median and the sampled standard deviation of WL for month j separately. After correlative relationship was established, the reverse procedure for reconstructing the WL time series can be achieved as

(7) $$\;h_{i,j}^{D}=I_{i,j}\times s_{j}+med\left(h_{j}\right)\;$$

where $h_{i,j}^{D}$ and $I_{i,j}$ represent the reconstructed WL and the drought index (i.e., SPI and GRACE-DSI) in year i and month j, respectively.

The TRMM precipitation, GRACE TWS, and their corresponding drought indices all represent the corresponding accumulations of the entire river basin; however, the spatial distributions of TRMM precipitation and GRACE TWS are not even. Therefore, a weighting factor is introduced to quantify the contribution of TRMM precipitation (or GRACE TWS) at each grid of the basin to the WL in the estuary, so that an optimal WL reconstruction is attained. The weighting factor can be obtained by the Pearson correlation coefficient (PCC) between the WL in the estuary and the two hydrological variables in each grid for the whole basin. The larger the PCC of the grid is, the stronger the relationship between the precipitation (TWS) at the grid and the observed WL is. In turn, this corresponds to a strong relationship between the precipitation (TWS) at this grid to the observed WL in the estuary. Hence, the basin weight-averaged precipitation and TWS are calculated as

(8) $$\;P_{i,j,k}^{w}=\frac{P_{i,j,k}\times \rho_{k}^{P}}{\sum \rho_{k}^{P}}\;$$

(9) $$\;S_{i,j,k}^{w}=\frac{S_{i,j,k}\times \rho_{k}^{S}}{\sum \rho_{k}^{S}}\;$$

where $P_{i,j,k}^{w}$ ($S_{i,j,k}^{w}$) are the weighted precipitation (TWS) at gridded location k in year i and month j, and $P_{i,j,k}$ ($S_{i,j,k}$) represent precipitation (TWS) at gridded location k in year i and month j. $\rho_{k}^{P}$ ($\rho_{k}^{S}$) is the PCC between the WL in the estuary and precipitation (TWS) at gridded location k. After the weighting of TRMM precipitation and GRACE TWS, the WL reconstruction procedure (containing replacements of the TRMM precipitation and GRACE TWS in their weighted form) was applied in both the MRB and YRB.

### 4.2. Assessment Schemes

Because the observed WL near the estuary represents the total input from the accumulated precipitation (TWS) of the entire river basin, the time series for the MRB and YRB are averaged or weight-averaged. To evaluate the performance of the reconstructed WL based on TRMM precipitation and GRACE TWS and their corresponding indices (i.e., TRMM-SPI and GRACE-DSI), the Pearson correlation coefficient (PCC), the root-mean-square error (RMSE), and the Nash–Sutcliffe model efficiency (NSE) coefficient were used. PCC is used to quantify the linear relationship between two variables, which can be calculated as

(10) $$\;PCC=\frac{\sum_{i=1}^{N}\left(X_{o}^{i}-\bar{X_{o}}\right)\left(X_{m}^{i}-\bar{X_{m}}\right)}{\sqrt{\sum_{i=1}^{N}{\left(X_{o}^{i}-\bar{X_{o}}\right)}^{2}}\sqrt{\sum_{i=1}^{N}{\left(X_{m}^{i}-\bar{X_{m}}\right)}^{2}}}\;$$

and RMSE represents the accuracy indicator for the estimate, which is defined as

(11) $$\;RMSE=\sqrt{\frac{\sum_{i=1}^{N}{\left(X_{m}^{i}-X_{o}^{i}\right)}^{2}}{N}}\;$$

The NSE, proposed by Nash and Sutcliffe [75], is designed to assess the hydrological models. It is given by

(12) $$\;NSE=1-\frac{\sum_{i=1}^{N}{\left(X_{m}^{i}-X_{o}^{i}\right)}^{2}}{\sum_{i=1}^{N}{\left(X_{o}^{i}-\bar{X_{o}}\right)}^{2}}\;$$

where $X_{o}$ and $\bar{X_{o}}$ represent the observation and its mean, and $X_{m}$ is the estimate. The NSE value ranges from −∞ to 1. The closer the NSE is to one, the more reliable the estimate is.

## 5. Results and Discussion

In this section, the WL reconstruction based on two hydrological variables (i.e., precipitation and GRACE TWS) and their corresponding derived indices (i.e., TRMM-SPI and GRACE-DSI) are presented. The impact of ENSO on the WL reconstruction is explained. The applied weighting procedure is also examined for whether a substantial improvement can be made to the WL reconstruction. Both internal and external assessments are conducted for accuracy comparisons among the aforementioned variables and indices. The internal assessment refers to the usage of three criteria (i.e., PCC, RMSE and NSE) described in Section 4.2 to evaluate the performance of the WL reconstruction based on hydrological variables and indices, whereas the external assessment refers to the usage of external data (i.e., another station time series; Dinh An in the MRB and Hukou in the YRB) to validate the applicability for WL prediction performance.

**Figure 3:**
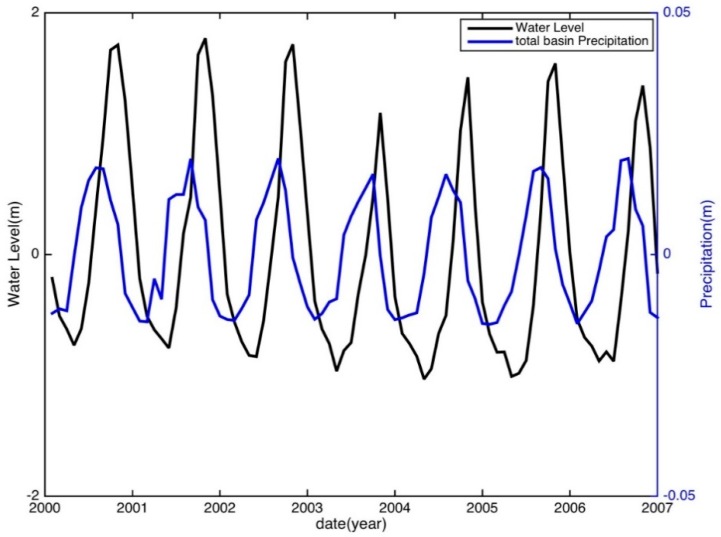
Time series of water level and basin-averaged TRMM precipitation in the MRB.

Note that a temporal lag between precipitation (TWS) and WL for some basins may exist, which is dependent upon the climate [76], the topography [77] and the hydrogeology [78] of the basin. A two-month time lag between TRMM precipitation and the WL was found (Figure 3), while no time lag between GRACE TWS and the WL was found in the MRB. In contrast, both TRMM precipitation and GRACE TWS are synchronous with the WL in the YRB on a monthly scale. This can be explained by the obvious differences in climate and hydrogeological conditions for the two basins. Therefore, the two-month time shift of the WL in MRB was applied before establishing the correlative relationship.

**Figure 4:**
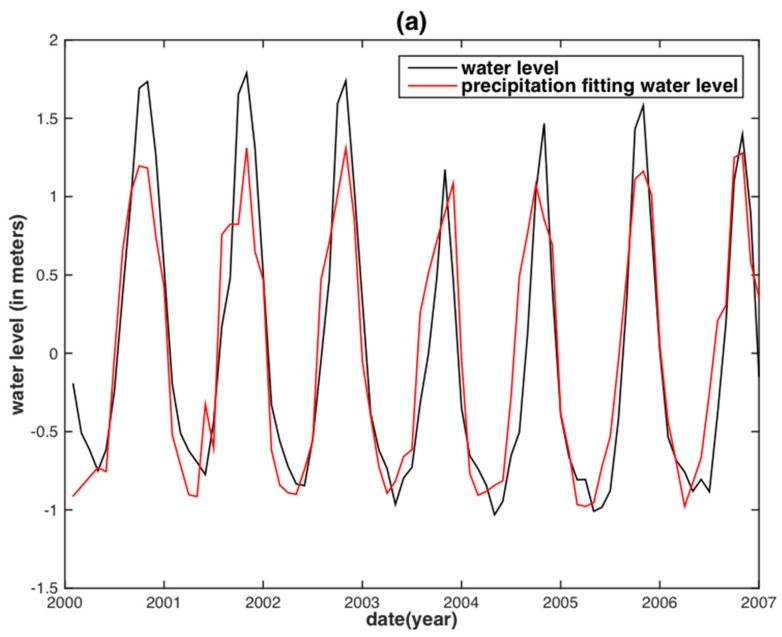

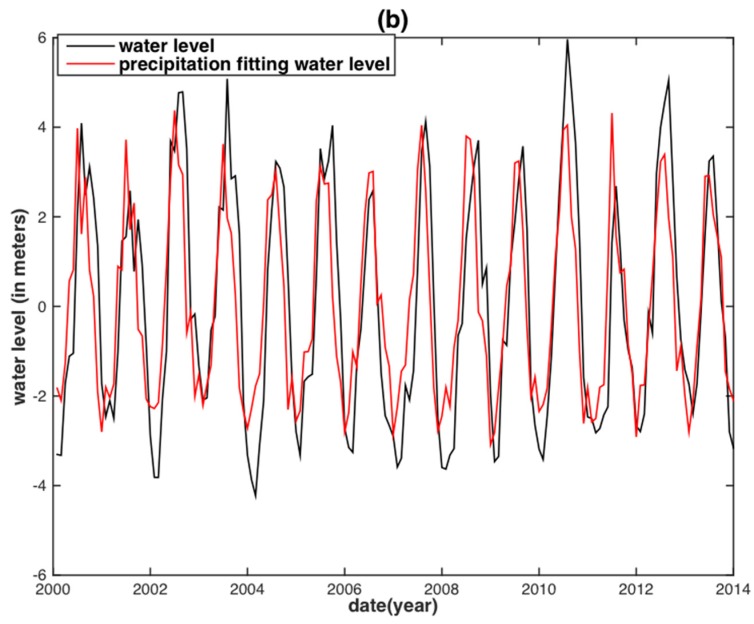
Water level reconstruction from basin-averaged TRMM precipitation at Vam Kenh station of the MRB (**a**) and at Datong station of the YRB (**b**).

**Figure 5:**
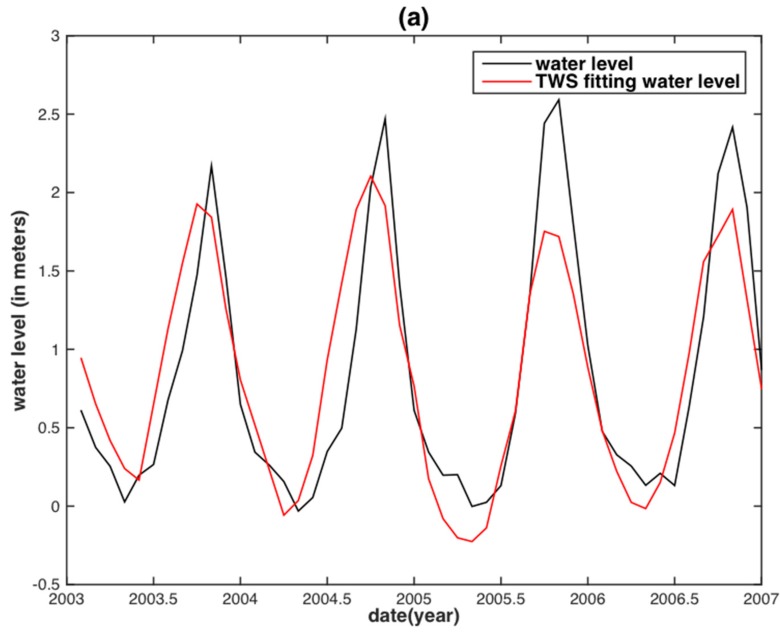

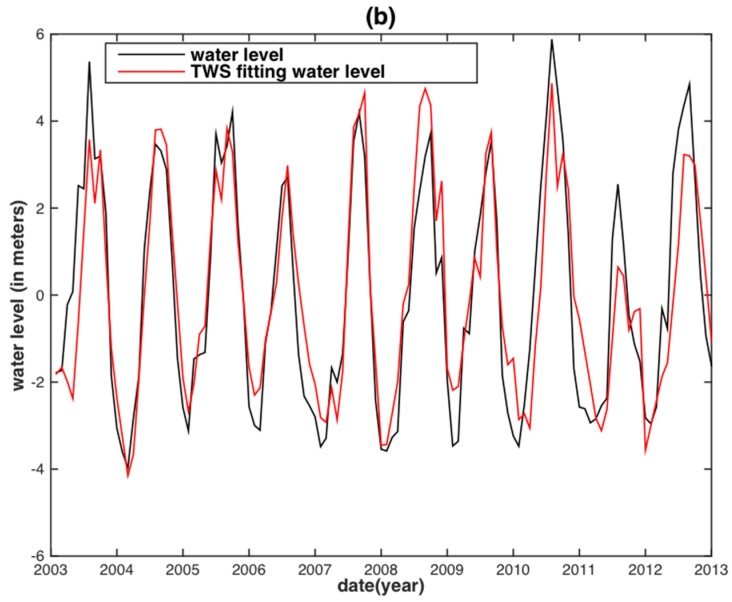
Water level reconstruction from GRACE TWS at Vam Kenh station of the MRB (**a**) and at Datong station of the YRB (**b**).

To assess the correlation of the internal assessment, the correlative relationships between the observed monthly WL and the two hydrological variables were obtained by linear regression (i.e., fitting with an offset plus slope). The regression would then be used to predict the WL in the two separate basins (Figure 4 and Figure 5). Both TRMM precipitation and GRACE TWS reconstructed WLs are in agreement with the observed WL. However, for the MRB, almost all WL peaks between the TRMM precipitation reconstructed WL and the observed WL displayed large discrepancies, except for the years 2003 and 2007 (Figure 4a). The GRACE TWS reconstructed WL shares both similar results with that of its precipitation counterpart. In addition, a time delay shorter than one month between the GRACE TWS reconstructed WL and observed WL is displayed (Figure 5a). For the YRB, the TRMM precipitation reconstructed WL has obvious underestimations occurring in the years of 2002, 2003, 2010 and 2012 (Figure 4b), whereas the GRACE TWS-derived WL is underestimated in the years of 2003, 2010, 2011 and 2012 (Figure 5b).

Given the above result, the apparent differences occur in the years 2000–2003 and 2004–2006 for the MRB and in the years 2002–2003 and 2010–2012 for the YRB, respectively. The discrepancies of these years may be attributable to the ENSO events (including El Niño and La Niña events) that occurred in 1998–2001 (very strong La Niña event), 2002–2003 (medium El Niño event), 2007–2008 (medium La Niña event), 2009–2010 (medium El Niño event) and 2010–2012 (medium La Niña event) based on the Sea Surface Temperature (SST) in the Niño 3.4 region (see Figure 3 from [79]). The El Niño (La Niña) events may reduce (increase) the precipitation and discharge in MRB but increase (reduce) the water in YRB [58,80,81].

The very strong 1998-2001 La Niña event is responsible for the TRMM precipitation reconstructed WL underestimation in the MRB during the period of 2000–2002. Both TRMM precipitation and GRACE TWS WL estimations of the YRB in the years 2003 and 2010–2012 are affected by the medium El Niño events in 2002–2003 and 2009–2010, respectively. Neither El Niño nor La Niña events are observed during the period of 2004–2006. Hence, the GRACE TWS reconstructed WL underestimation during this period may be caused by factors other than the climate.

**Figure 6:**
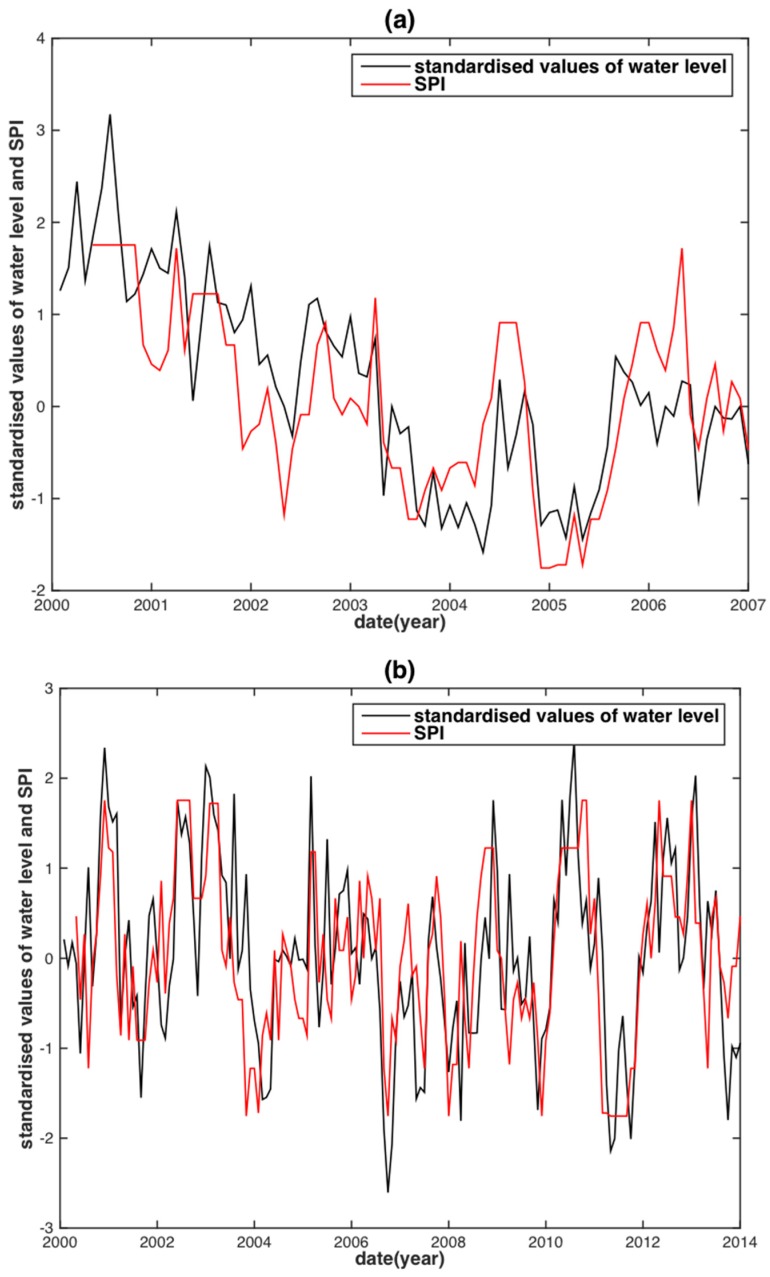

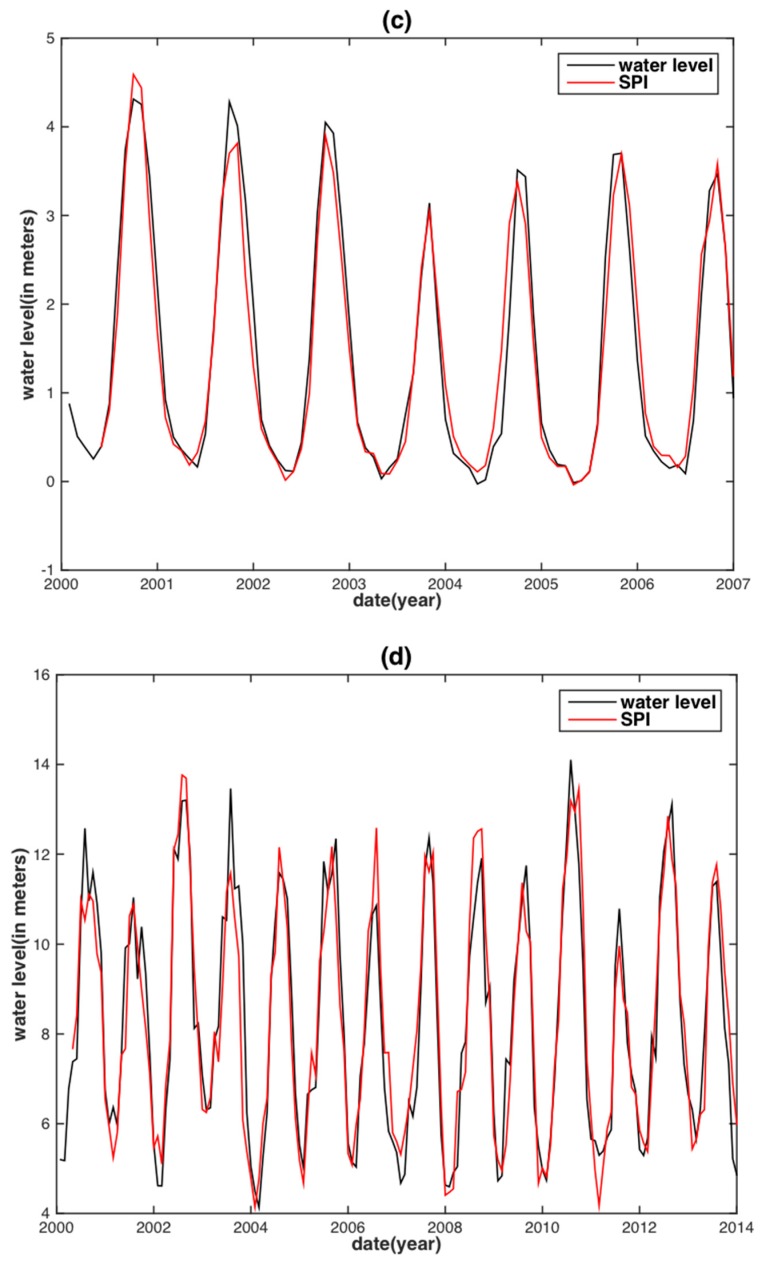
Time series of TRMM-SPI values against the standardized water level in the MRB (**a**) and the YRB (**b**); TRMM-SPI reconstructed water level against the observed water level in the MRB (**c**) and the YRB (**d**).

**Figure 7:**
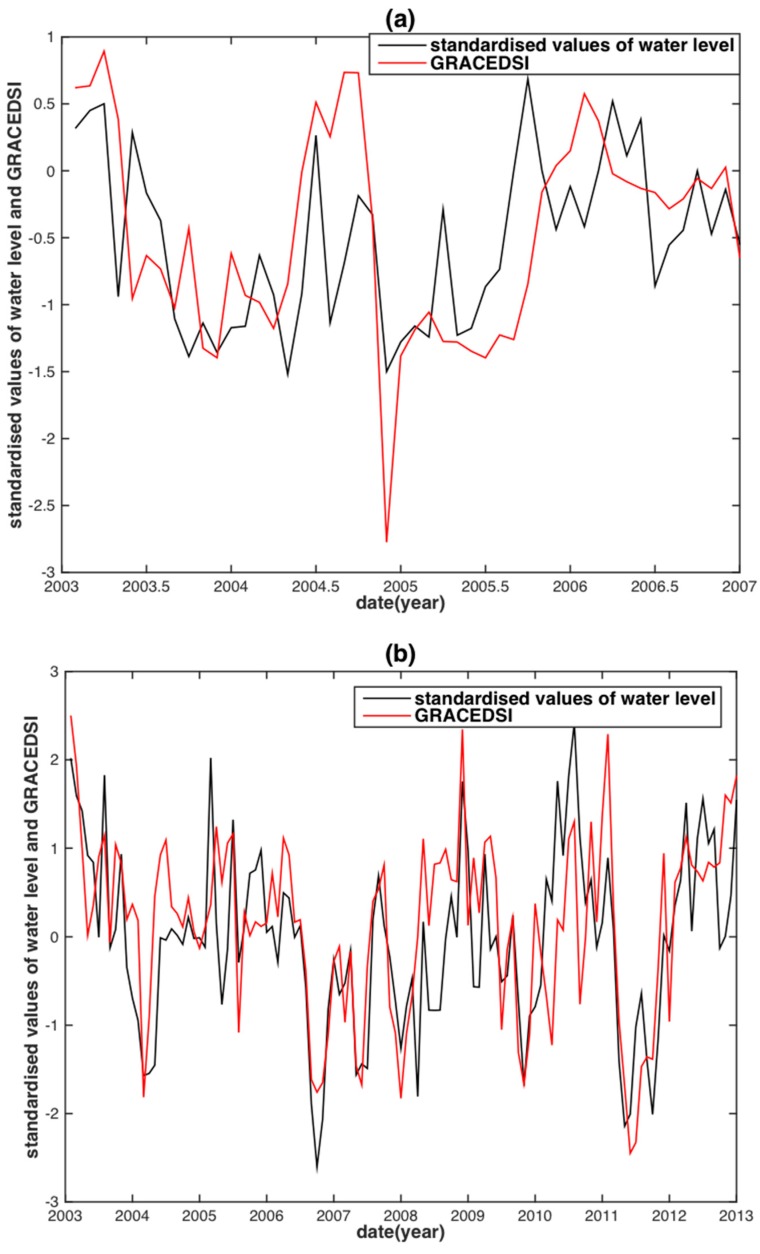

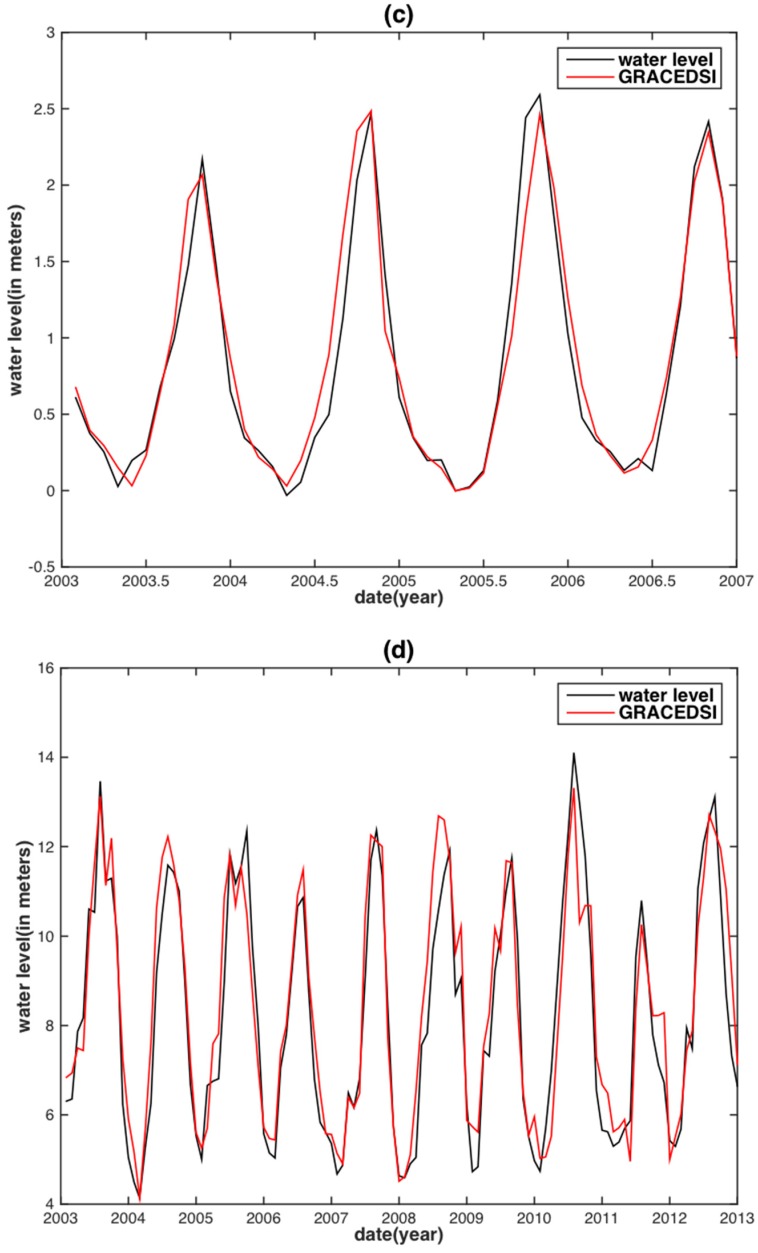
Time series of GRACE-DSI values against the standardized water level in MRB (**a**) and YRB (**b**); GRACE-DSI reconstructed water level against the observed water level in MRB (**c**) and YRB (**d**).

The TRMM-SPI shares the same tendency with the SWL in both the MRB and the YRB despite some minor differences (Figure 6a,b). The TRMM-SPI reconstructed WL for the two basins shows almost perfect agreement with their corresponding observed WL, except for several slight discrepancies of WL peaks (Figure 6c,d). Note that the best time scales of TRMM-SPI to reconstruct WL are five months for the MRB and four months for the YRB. This result shows a consistency with the findings from previous studies as described in Section 3.1. Contrary to the subjective choice for the best time scales of TRMM-SPI, no time scale is needed from GRACE-DSI, as it is simply calculated from the standardization formulae (i.e., Equation (5)). A similar performance of GRACE-DSI is achieved when compared to TRMM-SPI (Figure 7c,d).

Compared with the precipitation and TWS reconstructed WL in the MRB and the YRB (Figure 4 and Figure 5), the TRMM-SPI and GRACE-DSI reconstructed WL shows notable improvement (Figure 6c,d and Figure 7c,d). Almost all underestimations and overestimations of WL peaks diminished or even vanished in the TRMM-SPI and GRACE-DSI reconstructed results. This finding reveals that the WL reconstruction based on drought indices derived from hydrological variables can somehow bypass the influence of ENSO events. We speculate that this may also be attributable to basin-wide integrated information (i.e., Precipitation and TWS) and the standardization process in deriving the indices.

The above results are all built on the basin-averaged time series of the two hydrological variables and their corresponding derived indices. However, the spatial distribution of the precipitation and the TWS for the entire basin is uneven. The mean annual precipitation in the middle and lower YRB (i.e., 1600–1900 mm) is much larger than that in the upper area (270–500 mm) [57]. For the MRB, the precipitation pattern of its downstream, which is different from its upstream, is under the influence of monsoons and is also affected by the tropical cyclones coming from the east [82]. Therefore, a weighting factor should be able to improve the WL reconstruction accuracy. The PCC between the precipitation (TWS) and the WL in the two basins was adopted as relative weighting measure to recalculate the total basin-averaged precipitation (TWS) accumulation (hereafter called weight-averaged TRMM precipitation and weight-averaged GRACE TWS, respectively) (see Section 3.1).

**Figure 8:**
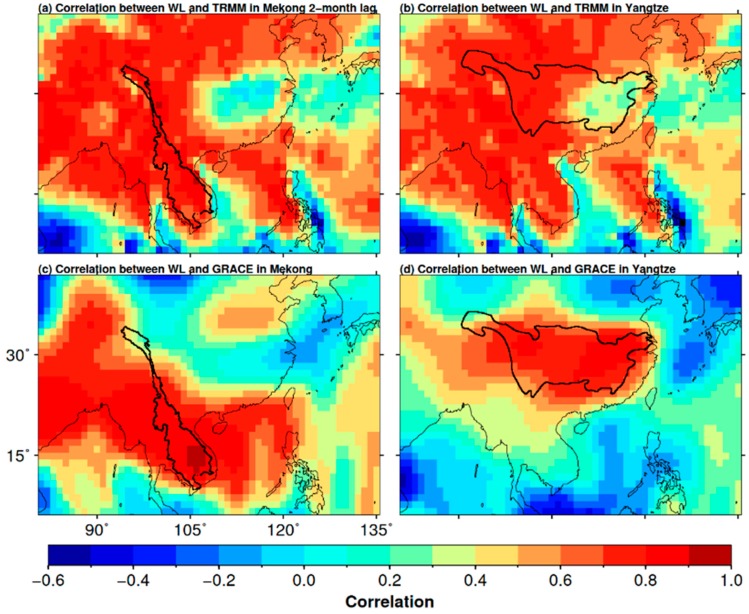
Correlation between remotely sensed TRMM precipitation and the observed water level in the MRB (**a**) and the YRB (**b**); The correlation between GRACE TWS and the observed the water level in the MRB (**c**) and the YRB (**d**). Note that the left-top panel (**a**) represents that the precipitation time series shifts two months forward before correlating with the observed water level.

Figure 8 displays the correlation between the TRMM precipitation and the observed WL, and the correlation between the GRACE TWS and the observed WL in the MRB and the YRB. Note that due to the two-month time shift between TRMM precipitation and the WL in the MRB has been applied. Precipitation in every gridded location is highly correlated (i.e., all PCC values are close to 0.8) with WL in MRB (Figure 8a). A similar pattern is observed for the TWS, except for the uppermost part of the basin (Figure 8c).

For the YRB, the correlation between TRMM precipitation and the WL ranges from 0.3 in the lower to 0.8 in the upper basin (Figure 8b). The lower YRB is affected by the EASM, which has significant impact on rainfall [50,56] and may cause the changes of rainfall in terms of start time, duration and intensity. This finding may be responsible for the relatively low correlation between precipitation and the WL in the lower basin in addition to intensive human activities in the lower YRB. In general, the TWS is highly correlated with the WL for the entire YRB (Figure 8d). Therefore, we anticipate the relationship of precipitation (with its derived TRMM-SPI) or TWS (with its derived GRACE-DSI) with the WL is strengthened by the weighting factor based on correlations, and hence, the performance of WL reconstruction should be improved.

**Figure 9:**
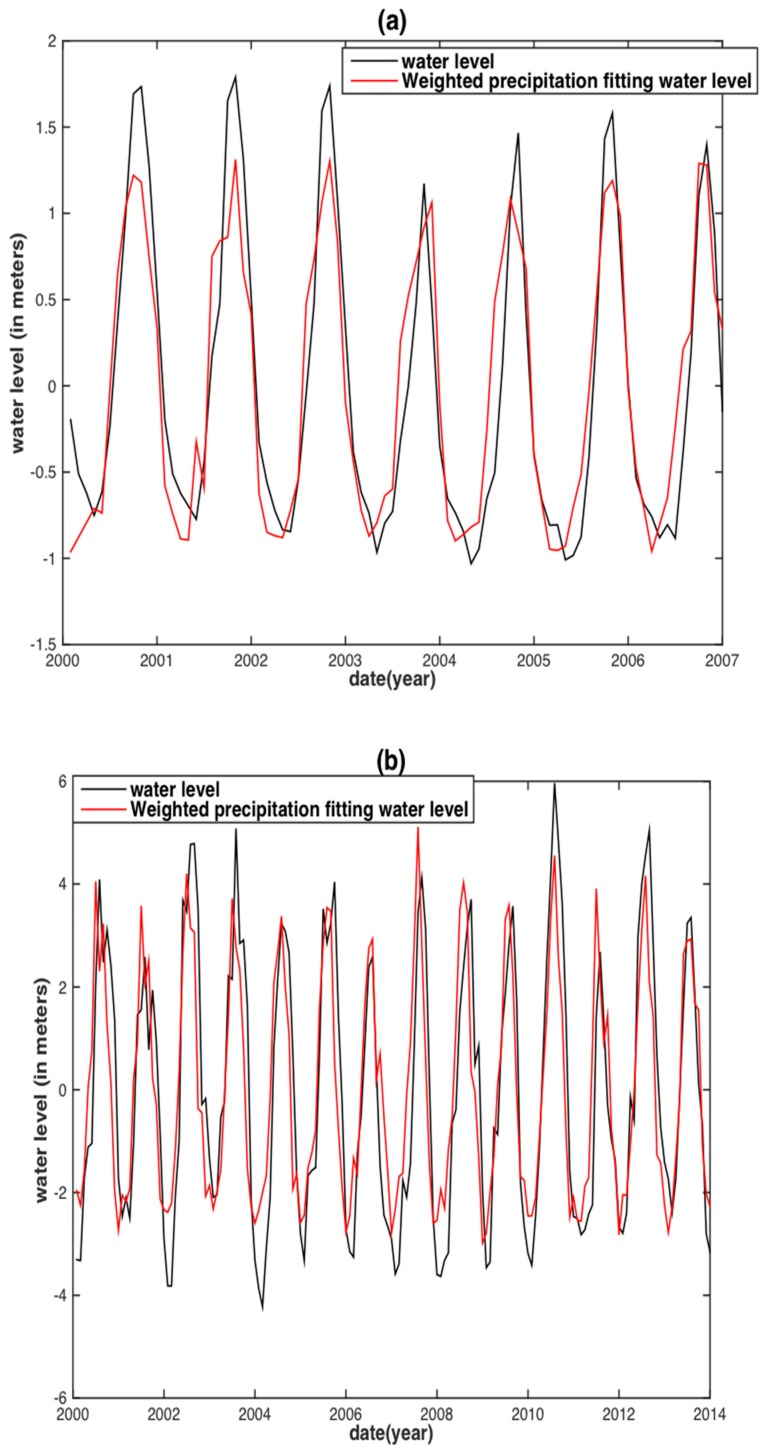

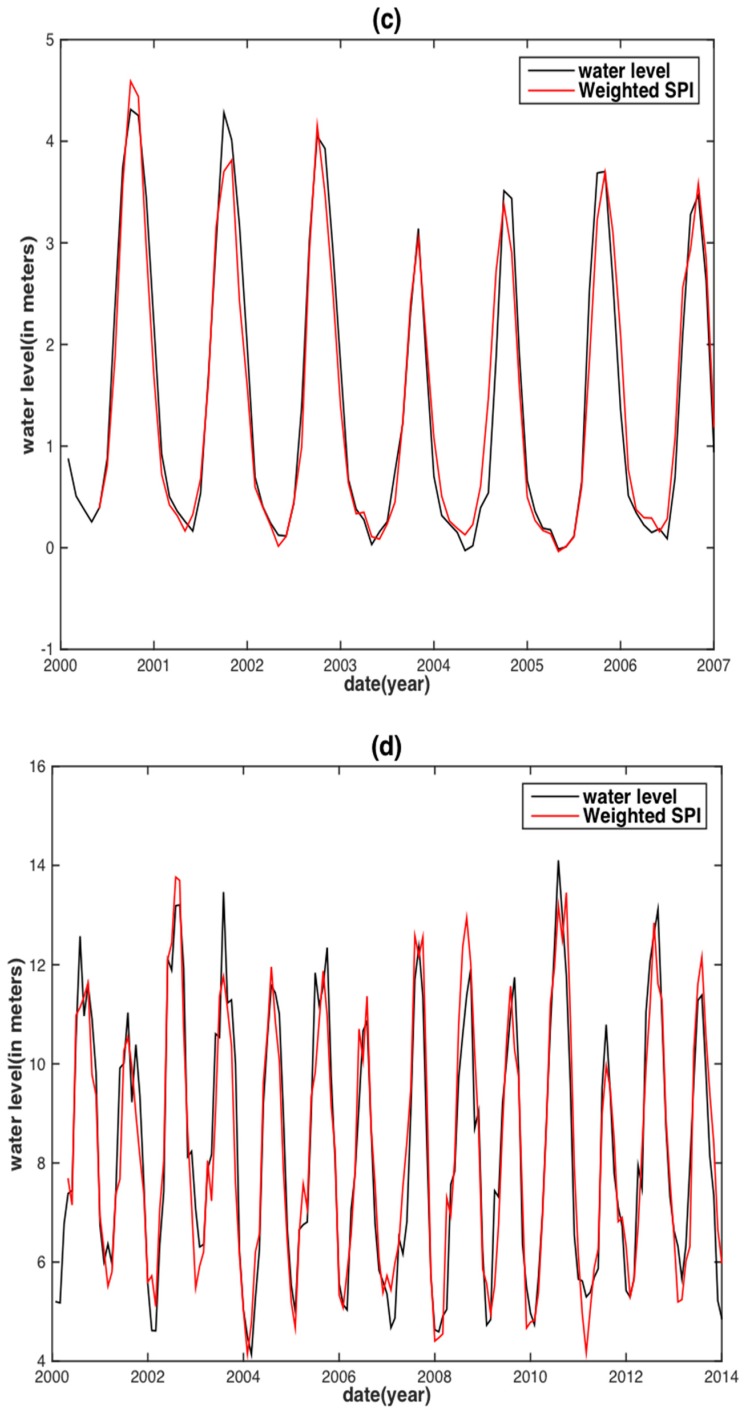
Water level reconstruction based on weight-averaged TRMM precipitation against the observed water level in the MRB (**a**) and the YRB (**b**) and weight-averaged TRMM-SPI reconstructed water level against the observed water level in the MRB (**c**) and the YRB (**d**).

**Figure 10:**
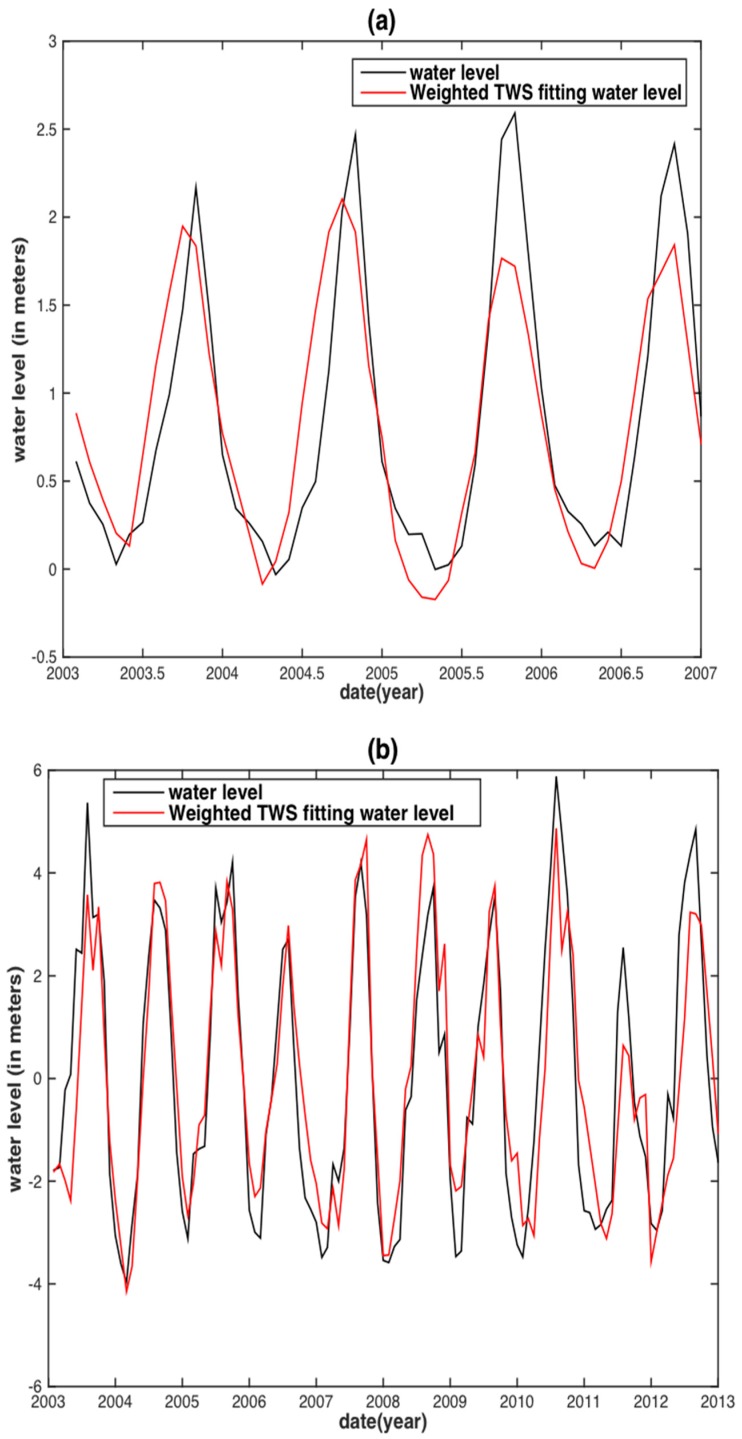

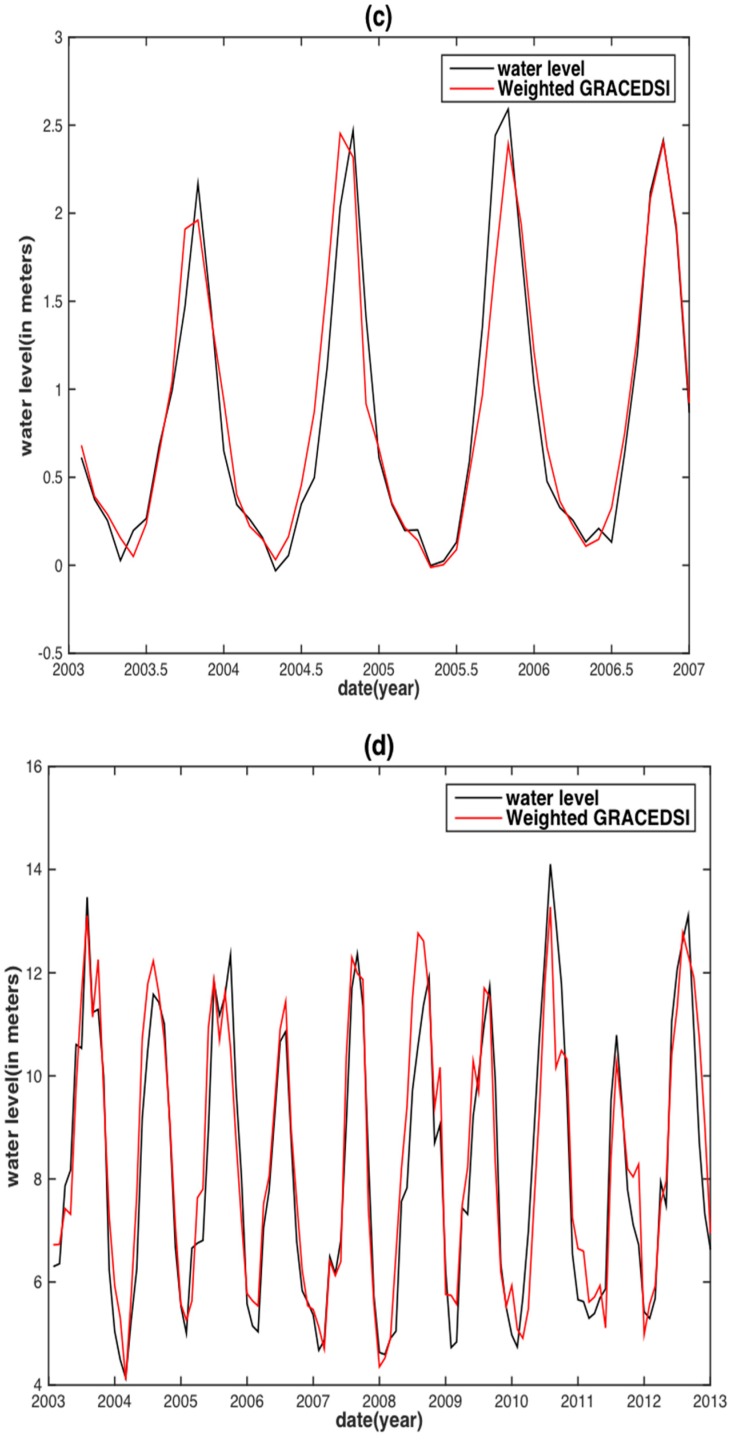
Water level reconstruction based on weight-averaged GRACE-derived TWS against the observed water level in the MRB (**a**) and the YRB (**b**) and weight-averaged GRACE-DSI reconstructed water level against the observed water level in the MRB (**c**) and the YRB (**d**).

After the weighting procedure was applied, the precipitation and TWS were weighted and later converted into weighted TRMM-SPI and weighted GRACE-DSI for reconstructing the WL in the MRB and the YRB based on the same procedure (Figure 9 and Figure 10). However, the differences between the unweighted and weighted procedures are not apparent in the figures. Therefore, both assessment schemes were adopted to recognize these differences, including three criteria: PCC, RMSE and NSE.

Table 1 shows the evaluation of two types of variables: traditional remote sensing variables (i.e., NDVI and LST), and remotely sensed hydrological variables (i.e., TRMM precipitation and GRACE TWS related variables). In general, the performance of the TRMM precipitation and GRACE TWS related variables are better than the traditional remote sensing variables in both the MRB and the YRB. This reveals that water balance components (i.e., precipitation and TWS) are better predictors. Furthermore, the reconstructed WL derived from precipitation related variables performs better than TWS derived variables in the MRB, although it is reverse in the YRB. This result indicates that precipitation (TWS) has a larger (smaller) contribution to WL in the MRB than the YRB. This finding highlights the geographic differences between the MRB and the YRB in terms of monsoonal climate (i.e., both tropics and subtropics) that govern precipitation; latitudinal (i.e., MRB) and longitudinal (i.e., YRB) direction and topography that govern the pathway and the speed of flow; and underground hydro-geologic properties that govern the storage-runoff relationship. 

Compared with unweighted variables and indices, the performances of their weighted counterparts are shown to have a substantial improvement, except for the weighted precipitation inferred WL in the MRB. This minor degradation may be ascribed to the time lag between precipitation and the WL since all other combinations without a time lag were improved by applying the weighting procedure. This improvement varies from 0.2% (TRMM-SPI in Mekong) to 4.7% (precipitation in Yangtze) for the increase in PCC, from 0.7% (GRACE-DSI in Yangtze) to 9.3% (precipitation in Yangtze) for the decrease in RMSE, and from 6.2% (GRACE-DSI in Yangtze) to 9.7% (precipitation in Yangtze) for the increase in NSE. In addition, the weighting procedure has the largest impact on precipitation-inferred WL in the YRB, which probably results from its least accurate reconstruction performance compared with other results. This indicates that the less accurate the performance is, the more the improvement it yields after the weighting procedure. We posit that the weighting procedure is a useful method for improving the accuracy of WL reconstructions.

We conclude that precipitation and TWS can compute the WL accurately as a result of PCC values of more than 0.81, RMSE values of less than 1.53 m, and NSE values of over 0.65. After standardization, the TRMM-SPI and GRACE-DSI perform better than that of direct precipitation and TWS (i.e., PCC > 0.94, RMSE < 0.88 m, and NSE >0.88). Finally, all results are improved by the weighting procedure. Overall, the best WL estimations in both the MRB and the YRB are derived from a weighted TRMM-SPI, with PCCs of 0.98 and 0.95, RMSEs of 0.19 m and 0.85 m, and NSEs of 0.95 and 0.85 for the MRB and the YRB, respectively.

To externally assess the applicability of our method, a similar procedure was applied to predict the WL at the Dinh An (Hukou) station in the MRB (YRB) based on the reconstructed relationships. For the MRB, the results in both the Dinh An and Vam Kenh stations are close in terms of their PCC and NSE values, but the results in Dinh An are slightly larger than that of Vam Kenh (Table 1). The external assessment in the YRB also shows a similar performance. Overall, our result is still shown to have a good performance (Table 1). Our approach can be useful to not only the WL reconstruction in gauged stations, but also for reconstructing the WL of ungauged stations via the data of other gauged stations in surrounding regions.

## 6. Conclusions

Contrary to the traditional WL estimation based on remote sensing variables (i.e., NDVI and LST), a method of reconstructing WL was investigated in the MRB and the YRB using the remotely sensed precipitation, TWS and their corresponding drought indices (i.e., TRMM-SPI and GRACE-DSI) on a monthly scale. We found that the total basin-averaged precipitation and TWS matched well with the observed WL in both river basins. Their corresponding indices derived through standardization can further improve the accuracy of WL estimations because the standardized procedure can bypass the influence of ENSO on the precipitation and TWS of the two basins.

For further improvement to our model, a weighting factor (i.e., PCC between precipitation (TWS) and WL), was also introduced to calculate the weight-averaged precipitation (TWS) of the total basin. The weighted TRMM-SPI obtained the best reconstructed WL with PCC values of 0.98 and 0.95, RMSE values of 0.19 m and 0.85 m, and NSE values of 0.95 and 0.85 for the MRB and the YRB, respectively. The results in the two basins derived from the weighted GRACE-DSI also display a good performance, with PCCs of 0.96 and 0.95, RMSEs of 0.21 m and 0.88 m, and NSEs of 0.93 and 0.89, respectively. Since the standardization and weighting procedures perform well for the two hydrological variables and their corresponding indices in the two basins, it is likely to be applicable for other variables (e.g., evapotranspiration) and indices (i.e., ENSO and monsoon indices) for more accurate WL reconstructions in other large basins.

Note that different hydrological variables may have different performances in different river basins, as the existence and length of the hysteretic relationship between hydrological variables and WL depend on the hydro-climatic and hydrographic conditions, not to mention different conditions for the up-, mid-, and down-stream segments of each basin. A comprehensive assessment for different river basins is necessary to further validate the applicability of the presented method in the near future. In addition to testing an improved temporal resolution of remotely sensed hydrological variables, our proposed method can be further extended by analyzing the hysteresis relationship that is affected by topography, climate and hydrogeology for different river basins while considering different combinations of hydrological variables for improving the WL reconstruction and estimation methodology.

## Figures and Tables

**Table 1:** Performance of the reconstructed water level from different indices at Vam Kenh (Datong) station in the MRB (YRB) and the predicted water level at Dinh An (Hukou) station in the MRB (YRB), respectively, against the observed water level. The predictions for Dinh An in the MRB and for Hukou in the YRB serve as the external assessment. The Dinh An (Hukou) prediction means the WL of Dinh An (Hukou) was predicted based on the reconstructed relationships between the WL from the Vam Kenh (Datong) station and abovementioned variables.

Basin	Data	PCC	RMSE (m)	NSE
Mekong (Vam Kenh)	NDVI	0.838	0.467	0.703
	LST	0.881	0.405	0.776
	P	0.909	0.355	0.827
	Weighted P	0.909	0.356	0.826
	TRMM-SPI	0.975	0.195	0.949
	Weighted TRMM-SPI	0.976	0.193	0.950
	TWS	0.874	0.381	0.764
	Weighted TWS	0.879	0.374	0.772
	GRACE-DSI	0.961	0.341	0.922
	Weighted GRACE-DSI	0.964	0.210	0.928
Yangtze (Datong)	NDVI	0.837	1.411	0.700
	LST	0.819	1.479	0.671
	P	0.805	1.528	0.648
	Weighted P	0.843	1.385	0.711
	TRMM-SPI	0.941	0.878	0.884
	Weighted TRMM-SPI	0.945	0.850	0.891
	TWS	0.905	1.110	0.819
	Weighted TWS	0.914	1.057	0.836
	GRACE-DSI	0.945	0.881	0.886
	Weighted GRACE-DSI	0.946	0.875	0.887
Mekong (Dinh An prediction)	NDVI	0.835	0.787	0.677
	LST	0.900	0.645	0.783
	P	0.907	0.582	0.823
	Weighted P	0.909	0.576	0.826
	TRMM-SPI	0.974	0.319	0.948
	Weighted TRMM-SPI	0.976	0.307	0.952
	TWS	0.880	0.578	0.774
	Weighted TWS	0.882	0.572	0.779
	GRACE-DSI	0.953	0.370	0.907
	Weighted GRACE-DSI	0.959	0.347	0.919
Yangtze (Hukou prediction)	NDVI	0.832	1.827	0.693
	LST	0.821	1.882	0.674
	P	0.816	1.903	0.667
	Weighted P	0.850	1.737	0.722
	TRMM-SPI	0.942	1.122	0.884
	Weighted TRMM-SPI	0.944	1.099	0.889
	TWS	0.900	1.455	0.810
	Weighted TWS	0.911	1.379	0.829
	GRACE-DSI	0.946	1.122	0.887
	Weighted GRACE-DSI	0.947	1.117	0.888
